# Typographical Errors

**Published:** 1848-01

**Authors:** 


### TYPOGRAPHICAL ERRORS.

We are sorry to find so many typographical errors in our first number. This may have been partly our fault—for we have not been in the habit of much proof-reading—yet correspondents must not expect us to make good english out of hieroglyphics. Several have complained and not without cause of the bad sense which such errors have made in the reading of their articles. We hope hereafter to improve in this, as well as in other respects, nntil the Register shall appear in perfect dress, within and without.—C. Ed.

